# iReenCAM: automated imaging system for kinetic analysis of photosynthetic pigment biosynthesis at high spatiotemporal resolution during early deetiolation

**DOI:** 10.3389/fpls.2023.1093292

**Published:** 2023-04-21

**Authors:** Veronika Balakhonova, Tereza Dobisova, Zuzana Benedikty, Klara Panzarova, Jaromir Pytela, Radka Koci, Ioannis Spyroglou, Ingrid Kovacova, Dominique Arnaud, Jan Skalak, Martin Trtilek, Jan Hejatko

**Affiliations:** ^1^ CEITEC - Central European Institute of Technology, Masaryk University, Brno, Czechia; ^2^ National Centre for Biomolecular Research, Faculty of Science, Masaryk University, Brno, Czechia; ^3^ Photon Systems Instruments, Drasov, Czechia

**Keywords:** de-etiolation, chlorophyll biosynthesis, fluorescence, iReenCAM, cytokinins, ethylene, *Arabidopsis*

## Abstract

Seedling de-etiolation is one of the key stages of the plant life cycle, characterized by a strong rearrangement of the plant development and metabolism. The conversion of dark accumulated protochlorophyllide to chlorophyll in etioplasts of de-etiolating plants is taking place in order of ns to µs after seedlings illumination, leading to detectable increase of chlorophyll levels in order of minutes after de-etiolation initiation. The highly complex chlorophyll biosynthesis integrates number of regulatory events including light and hormonal signaling, thus making de-etiolation an ideal model to study the underlying molecular mechanisms. Here we introduce the iReenCAM, a novel tool designed for non-invasive fluorescence-based quantitation of early stages of chlorophyll biosynthesis during de-etiolation with high spatial and temporal resolution. iReenCAM comprises customized HW configuration and optimized SW packages, allowing synchronized automated measurement and analysis of the acquired fluorescence image data. Using the system and carefully optimized protocol, we show tight correlation between the iReenCAM monitored fluorescence and HPLC measured chlorophyll accumulation during first 4h of seedling de-etiolation in wild type *Arabidopsis* and mutants with disturbed chlorophyll biosynthesis. Using the approach, we demonstrate negative effect of exogenously applied cytokinins and ethylene on chlorophyll biosynthesis during early de-etiolation. Accordingly, we identify type-B response regulators, the cytokinin-responsive transcriptional activators ARR1 and ARR12 as negative regulators of early chlorophyll biosynthesis, while contrasting response was observed in case of EIN2 and EIN3, the components of canonical ethylene signaling cascade. Knowing that, we propose the use of iReenCAM as a new phenotyping tool, suitable for quantitative and robust characterization of the highly dynamic response of seedling de-etiolation.

## 1 Introduction

De-etiolation is one of the most dramatic stages in the plant life cycle, leading to a substantial re-arrangement of plant developmental program and a switch from heterotrophic to autotrophic metabolism. Light is the main trigger of de-etiolation, associated with inhibition of hypocotyl elongation, expansion and unfolding of the cotyledons and synthesis of individual components of the photosynthetic apparatus and photoprotective pigments (Casal et al., 2003; Chen et al., 2004). During de-etiolation, the plants must solve a problem of balancing the chlorophyll biosynthesis and assembly of the light-harvesting complexes, as the chlorophyll biosynthesis intermediates and the free chlorophyll itself can lead to harmful singlet oxygen formation due to photooxidation (Reinbothe et al., 1996). Furthermore, the chlorophyll biosynthesis is a part of the tetrapyrolle metabolism, including production of heme, siroheme and phycobillins, thus affecting the light signaling due to production of phytochromobilins, the cofactors of light-responsive phytochromes. As a consequence, the light-induced chlorophyll biosynthesis is tightly controlled by a number of regulatory components [reviewed in (Kobayashi and Masuda, 2016)].

Chlorophyll biosynthesis is a multistep metabolic pathway taking place in plastids. Briefly, protochlorophyllide, the chlorophyll precursor is synthesized in the darkness from the L-Glutamate and tRNA^Glu^ leading (through number of steps) to formation of 5-aminolevulonic acid (ALA), the universal precursor for all tetrapyrroles. Another important intermediate produced downstream the pathway is protoporphyrin IX, the precursor for the synthesis of both heme, allowing production of phytochromobilin, and protochlorophyllide, being converted upon illumination to chlorophyll [for more detailed description see (Tanaka et al., 2011; Brzezowski et al., 2015; Kobayashi and Masuda, 2016)].

Phytochrome-mediated light signaling controls the expression of genes encoding several enzymes involved in the chlorophyll biosynthesis. Based on their responsiveness to light and circadian rhythm, the genes coding for chlorophyll metabolism enzymes were categorized into four clusters (Matsumoto et al., 2004). Genes from the cluster 1 were found to be repressed in the etiolated seedlings but promptly upregulated by light as soon as 1 h after irradiation, reaching their nearly-maximal levels at around 3 h after light-mediated de-etiolation stimulation (Matsumoto et al., 2004; Kobayashi and Masuda, 2016). The cluster 1 genes include *GLUTAMYL-tRNA REDUCTASE (HEMA1)*, encoding ferrochelatase involved in ALA synthesis, one of the rate-limiting steps of the entire tetrapyrrole metabolic pathway. Cluster 2 comprises the genes from the earlier to the later steps, but in the contrast to genes from cluster 1, cluster 2 is primarily not affected by circadian control. Genes belonging to cluster 3 are not regulated neither by light nor circadially. Finally, the cluster 4 is formed by *PROTOCHLOROPHYLLIDE OXIDOREDUCTASE A* (*PORA)* and *PORB* only. Transcripts of both genes accumulate in the darkness and decrease upon illumination (Kobayashi and Masuda, 2016).

However, despite a rather prompt upregulation of chlorophyll biosynthetic genes, the chlorophyll formation starts much faster in the de-etiolating seedlings, leading to significant chlorophyll accumulation in order of minutes after irradiation [(Virgin et al., 1963), this work]. This is the result of photocatalysis mediated by light-dependent PORs, unique enzymes directly activated by light. The PORs form a complex with dark-synthesized protochlorophyllide and NADPH and oligomerize, leading to formation of paracrystalline structures in the etioplast membranes called prolamellar bodies (Heyes et al., 2021; Zhang et al., 2021). Upon irradiation, the protochlorophyllide in the complex acts as both the substrate and the light-sensing chromophore, initiating its own C17-C18 double bond reduction in the active site of PORs [(Heyes et al., 2021) and references there in]. The light absorption by the POR-protochlorophyllide-NADPH complex and the following electron and proton transfer between the NADPH and the protochlorophyllide tetrapyrrole ring takes place in order of ns to µs, representing the very first event in the plant de-etiolation and triggering an entire regulatory cascade initiating seedling de-etiolation (Belyaeva and Litvin, 2014; Heyes et al., 2021).

Cooperatively with light, hormones contribute to the regulation of the chlorophyll biosynthetic pathway [reviewed in (Liu et al., 2017)]. Cytokinins were shown to upregulate the ALA biosynthesis via upregulating the amount of tRNA^Glu^ in plastids as well as levels of glutamyl-tRNA in greening cucumber cotyledons (Masuda et al., 1994). Similarly, higher protochlorophyllide formation was observed in lines with upregulated endogenous cytokinin levels due to deficiency in cytokinin degradation (Hedtke et al., 2012). Both endogenous cytokinins upregulation as well as exogenous cytokinins application was found to activate expression of number of chlorophyll biosynthetic genes including *HEMA1*, which has shown to be direct target of cytokinin signaling (Tanaka et al., 2011; Hedtke et al., 2012; Cortleven and Schmulling, 2015; Cortleven et al., 2016; Kobayashi and Masuda, 2016). In addition to cytokinins, de-etiolation is under control of another important plant growth regulator ethylene, acting as negative regulator of protochlorophyllide formation, but positive factor of cotyledon greening, able to rescue the *cop*-like phenotypes in etiolated *Arabidopsis* (Zhong et al., 2009; Zhong et al., 2010).

Here we present a new tool, iReenCAM (*i*nducible g*Reen*ing *CAM*era), which was designed to accurately monitor chlorophyll dynamics at early stages of de-etiolation on a minute-scale after illumination of etiolated *Arabidopsis* seedlings. Our fluorescence measurement data obtained in both wild type and chlorophyll biosynthesis-deficient mutant lines significantly correlate with the HPLC-based quantification of chlorophyll levels, thus establishing the iReenCAM as a sensitive non-invasive tool with high spatial and temporal precision. To illustrate its utility, we compared responses of *Arabidopsis* seedlings to exogenous cytokinin and ethylene treatment, two important plant hormones previously shown to affect chlorophyll metabolism. Finally, we tested several *Arabidopsis* mutant lines deficient in the components of cytokinin and ethylene signaling, which led to the identification of essential components of the multistep phosphorelay affecting the chlorophyll status of the plant.

## 2 Materials and equipment

### 2.1 Plant material and growth conditions

Set of *Arabidopsis thaliana* mutants lacking chloroplastic FtsHs *ftsh1*, *ftsh2*, *ftsh5*, *ftsh8* ecotype Columbia (Col-0) (Duan et al., 2019) and *porB*, *porC* ecotype Landsberg erecta (Ler-1) (Frick et al., 2003) as well as corresponding wild-type, were kindly provided by Prof. Chanhong Kim (Shanghai Center for Plant Stress Biology, China). The cytokinin type-B ARR mutants [*arr1-3*, *arr12-1* (Mason et al., 2005); *arr10-5 (* Argyros et al., 2008 *)*), and ethylene-insensitive mutants [*ein2-1*, *ein3-1* (Roman et al., 1995)] were obtained from the Nottingham Arabidopsis Stock Centre.

*Arabidopsis* seeds (10-20 mg) were surface-sterilized by washing with 70% ethanol and then rinsed with sterile water several times. Seeds were sown in square Petri plates (120 x 120 x 17mm) containing 1.5% (w/v) Gelrite (Duchefa Biochemie, Haarlem, The Netherlands) following optimized sowing protocol as described further. In the case of the cytokinin and ethylene application, the appropriate hormone diluted from 5 mM stock solution was directly added to the cultivation media in final concentrations of 10, 100, 500 nM, and 1uM. For cytokinin treatments, 6-benzylaminopurine (BAP, Sigma) solution dissolved in dimethyl sulfoxide (DMSO, Sigma) was used. The seedlings were grown on 1.5% Gelrite containing BAP and DMSO as a control. The final DMSO concentration of 0.001% was kept in both control and BAP dilution. For ethylene treatments, water-soluble 1‐aminocyclopropane‐1‐carboxylic acid (ACC, Sigma) was applied.

Sowing and cultivation protocol was precisely optimized. Sowing grid with 3 rows divided in 5 areas (**Supplementary Figure 1**) was used for seed sowing with approximately 40 seeds sown per each area. The density of the seeds was optimized to minimize shading of neighboring plants and ensuring that all plants can be visualized. The seeding geometry was optimized in order to maximize the use of the homogeneously irradiated area and the light intensity did not drop below 70% of maximum light intensity. Each tested variant was sown in triplicate per plate with using randomised scheme and spatial separation of the individual areas.

After sowing, the plates were wrapped in aluminum foil and placed at 4˚C in darkness for 3 days. The induction of germination was done under white light (150 µmol photons m^−2^ s^−1^) for 1 h, after which seedlings were wrapped in aluminium foil and cultivated in vertical position in growth chambers for 4 days in the dark at 21 ˚C. The vertical position was used to visualize whole profile of the plant including root, hypocotyl and cotyledon. Prior measurement in iReenCAM the plates were unwrapped from the foil and in complete darkness were positioned into the pre-defined imaging position of the imager.

### 2.2 iReenCAM device

**Figure 1:**
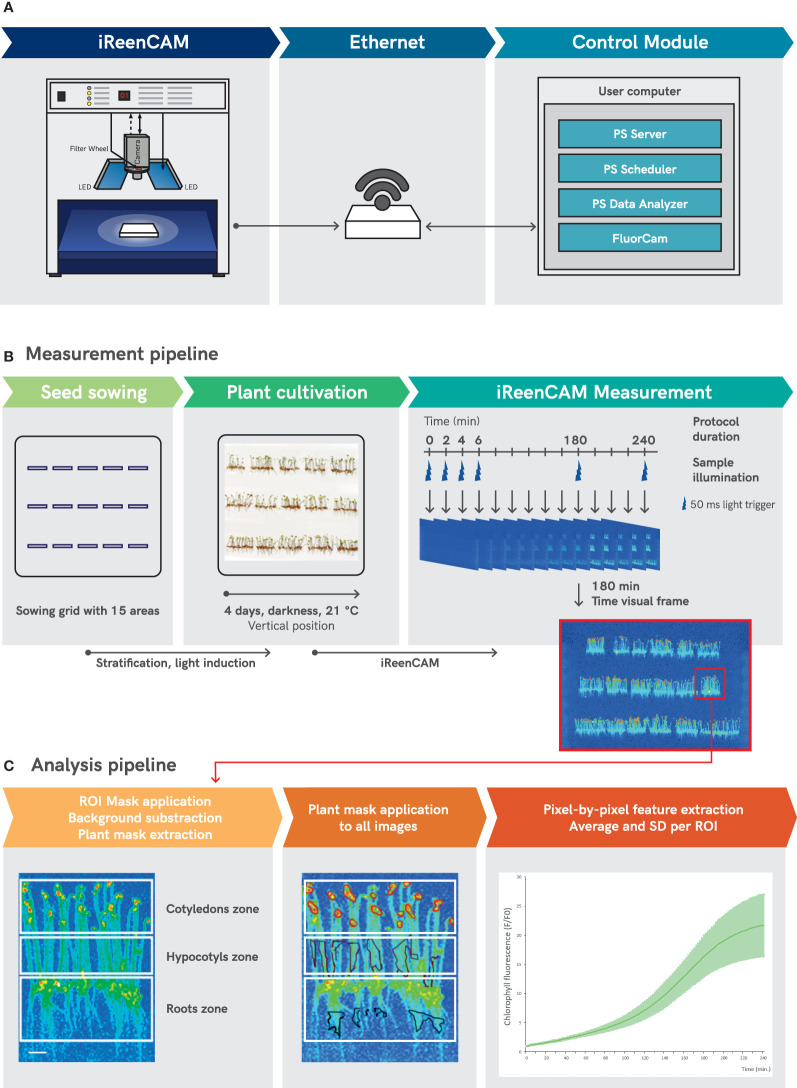
The scheme of iReenCAM measurement and analysis pipeline. **(A)** Comprehensive control module for automatic and programmable image acquisition and data management based on PlantScreen™ phenotyping SW toolbox organizes the operation of the entire iReenCAM system by controlling and synchronizing the HW operation with user defined measurement and analysis protocol. **(B)** Measurement pipeline consists of grid-defined seed sowing, followed by stratification, light induction and vertically oriented Petri plate cultivation. The measuring protocol allows dynamic measurements of the fluorescent images of a sample in 2-minute intervals for 4 hours in total, i.e. 120 measuring rounds. Time visual frame of representative false-color image of vertically oriented 4-day-old *Arabidopsis* seedlings acquired at time 180 min is used for ROI mask generation. **(C)** Mask defining ROI for cotyledon, hypocotyl and root zone is applied on time visual frame, further background subtraction is performed and plant mask is extracted. Next plant mask for the zone of interest of the seedlings is applied to images from all measured rounds and pixel by pixel fluorescence values for each ROI from all measured rounds is extracted. Finally, mean and SD values for given ROI (here for cotyledons only) was calculated (n=9) of raw data (fluorescence F) normalized to the mean fluorescence value at T0 (F0). Scale bars = 1 cm **(A)** and 0.25** **cm **(C)**.

A new iReenCAM imaging system (**Figure 1**) has been developed based on the enhanced version of the Closed GFPCam FC 800-C/1010GFP pulse amplitude modulated (PAM) chlorophyll fluorometer (PSI, Czech Republic). The iReenCAM consists of high sensitivity CCD camera TOMI-2 (resolution of 1360 x 1024 pixels, frame rate 20 fps and 16-bit depth) with integrated fully motorized and software-controlled filter wheel including appropriate filter sets and four super bright LED panels with dimension of 130 x 130 mm. Two opposite panels consisted of orange-red (618 nm) LEDs (used for PAM measuring flashes), two other consisted of blue (470 nm) LEDs (used for chlorophyll biosynthesis excitation and measurement). The LED panels provide uniform irradiance over an area 90 × 90 mm (data not shown).

The RG695 color-glass optical filter (Schott) in combination with short-pass interference filter (SP750), installed into a filter wheel in front of the CCD camera, was applied to detect the chlorophyll fluorescence. The filter set transmits the light in spectral region between 680–750 nm (discussed in **Figure S1**). The distance between the CCD camera and the sample was approximately 20 cm. The system is compact and light insulated allowing easy dark adaptation of an investigated sample.

Comprehensive control module for automatic and programmable image acquisition and data management based on PlantScreen™ phenotyping SW toolbox was used for automatic user defined data acquisition and image parameters extraction and analysis.

### 2.3 iReenCAM measurement and analysis

By the start of the experiment, the plate with four-days-old etiolated seedlings was in complete darkness manually placed into a light insulated cabinet of the iReenCAM device (**Figure 1A**). Further imaging and measuring of chlorophyll fluorescence was performed in the automatic regime using the PlantScreen™ phenotyping SW toolbox (PSI, Drásov, Czech Republic). The SW toolbox comprises *i.* PlantScreen™ Server application used as server service for main machine control, which executes experimental setups by client applications and provides interface to the SQL database; *ii.* PlantScreen™ Scheduler Client - program GUI between machine and user that connects to the server, displays actual system state, provides GUI for experiment setup and for control, and is used for visualization, planning and management of the individual experiments including protocol design; *iii.* PlantScreen™ Analyser software for automatic data storage in SQL database, data processing, data export and data re-analysis features (**Figure 1A**).

The iReenCAM protocol used in all experiments was designed using the PlantScreen™ Scheduler Client as follows. The protocol began with the exposure of etiolated seedlings to blue actinic light (470 nm) with light intensity of 240 µmol m^−2^ s^−1^ for 50 ms that refers to single measuring pulse providing 1,32 ×10^19^ photons per single light pulse. The integration time of the camera is synchronized with the light pulses. Thus, the time of sample illumination per single measuring round was 50 ms, too. The light pulse was followed by a dark period for subsequent 2 minutes that together was defined as one measuring round. The rounds ran automatically in sequence every 2 min for 4 hours. Single measuring protocol consisted of 120 measuring rounds (**Figure 1B**). The pulse of blue light generated from light-emitting diode (LED) panels initiated the chlorophyll formation in the etiolated seedlings and was in the same time used as excitation light to measure the fluorescence signal. The timing, duration, and intensity of the pulses were same for all measured rounds and were determined in the measuring protocol.

**Figure 2:**
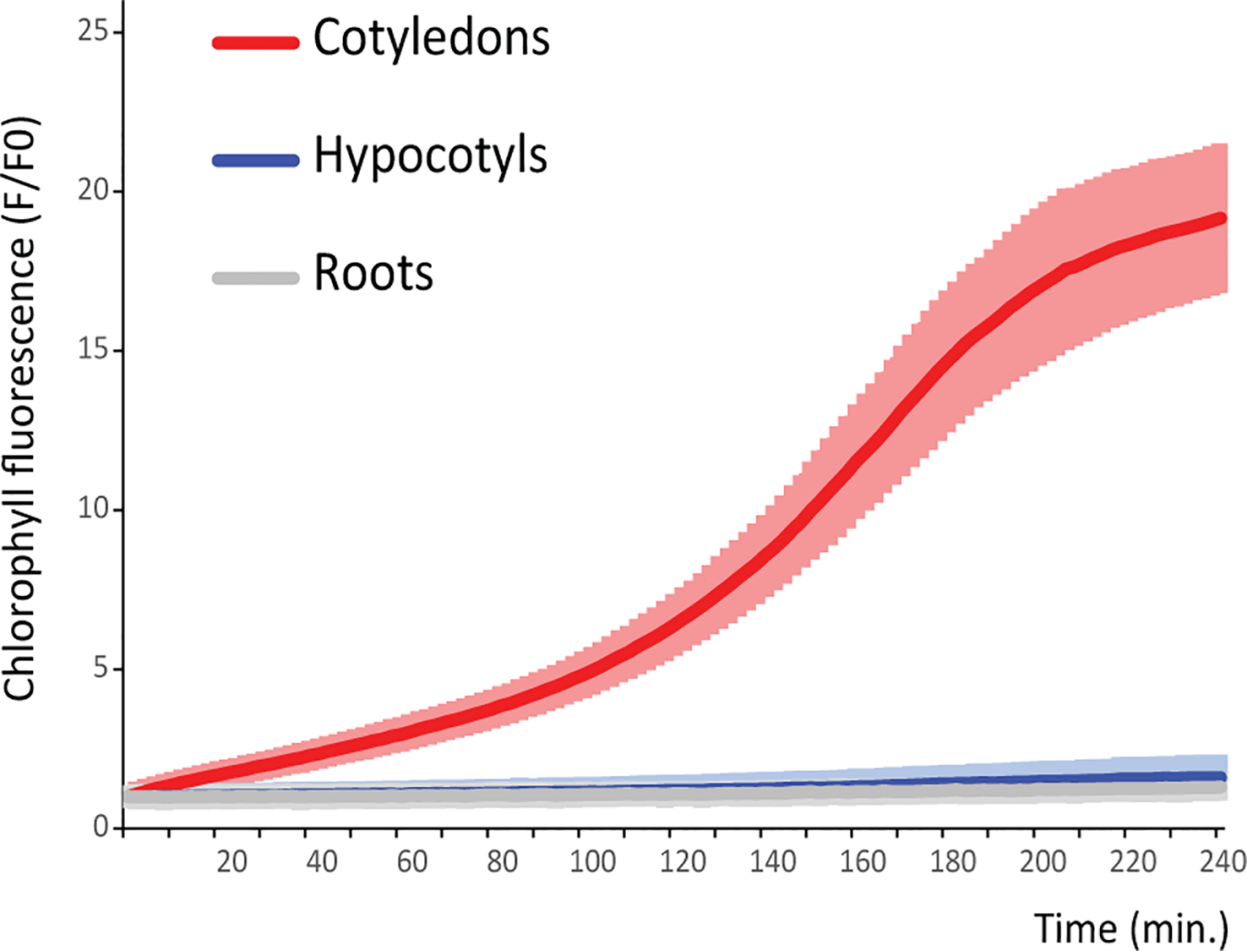
iReenCAM allows to quantify the chlorophyll accumulation with high spatial and time resolution. The region of interest for cotyledons, hypocotyls, and roots was manually selected by the separate setting of fluorescent intensity threshold for each seedling segment (see **Figure 1C**). The red curve shows typical results of chlorophyll accumulation kinetics in cotyledons. Mean values **±** SD, n=9 of raw data (fluorescence F) normalized to the mean fluorescence value at T0 (F0).

The PlantScreen™ Analyzer software was used to automatically process the raw data. The image analysis consisting of the automated ChlF feature extraction by mask application on image from measuring round acquired 3h after initiation of the measurement, i.e. frame with high contrast that is not in the saturation phase (**Figure 1C**). Further mask for region of interest (ROI) was defined for top part of the seedling referring to cotyledons (used for data in **Figure 1C**); for specific purpose also ROI for middle part of the seedlings referring to hypocotyls and bottom part referring to roots of the seedlings was used (see **Figure 2**). In the next step, background subtraction was performed resulting in plant mask generation (i.e. plants specific pixels) that was used for fluorescence level quantification by integrating pixel-by-pixel fluorescence values defined by plant mask for all measuring rounds acquired during one measuring protocol. Finally, the average numeric value of the chlorophyll fluorescence intensity (a.u.) for the given area was extracted from the 120 images acquired during one measuring protocol and used to quantify dynamics of chlorophyll biosynthesis.

### 2.4 HPLC

The protocol for chlorophyll extraction by methanol was employed. For each time point, seedlings were collected under dim green light, dissolved in methanol, disintegrated, and placed at -20°C for 24h. After, the samples were centrifuged at 15000 rpm for 20 min at 4°C. The supernatant was removed and evaporated. Shortly before HPLC analysis, the evaporated residue was redissolved in 200 µl of cooled methanol. Then the mixture was centrifuged at 15000 rpm for 10 min at 4°C to get the pure extract for further procedure. The HPLC analysis was performed according to Garcia-Plazaola and Becerril (1999). Kinetex RP C18 column (3.5 µm; 4.6x100 mm) was used for chromatography. The mobile phase consisted of two components: solvent A, acetonitrile:methanol:Tris buffer (0.1 M, pH 8.0) (42:1:7); and solvent B, ethyl acetate:methanol (17:8). Both solvents were of HPLC grade and degassed by ultrasonic bath for 30 min. The pigments were eluted using a linear gradient from 100% A to 100% B for the first 12 min with an isocratic elution with 100% B for 6 min. This step was continued with a 1 min linear gradient from 100% B to 100% A and isocratic elution with 100% A for the next 6 min for the column re-equilibration with solvent A before the next injection. The solvent flow rate was 1.2 mL/min, with working pressures below 1000 psi. The injection volume was 50 µl. The temperature of the column was 25°C. Chlorophyll pigment separation and identification were performed using individual pigment standards. Peaks were detected and integrated at 436 nm. Average concentrations of chlorophyll pigments (mg/g of Fresh Weight) were obtained from the three independent experiments.

### 2.5 Statistical analysis

Three independent experiments, each with at least three biological replicates were performed for statistical evaluation. The resulting graphs are based on the means and their standard deviations. The statistical significance of the difference between genotypes and treatments was assessed by the mixed models’ approach (Spyroglou et al., 2021). For the pairwise comparison of genotypes, the Benjamini-Hochberg Procedure correction method was used.

### 2.6 Steady-state fluorescence analysis at low-temperature (77 K)

Low temperature fluorescence spectra were measured in liquid nitrogen (77 K) with a CCD-array spectrometer SM-9000 spectrometer (Photon Systems Instruments, Czech Republic). The fluorescence was detected in 90° angle to the excitation beam of blue light (LED 420 nm). Samples of 4-days-old *Arabidopsis* seedlings (*WT Col-0*) were frozen in liquid nitrogen in the similar timepoints as for HPLC. Samples were placed on the metal spoon and immersed in the measuring cuvette pre-chilled by liquid nitrogen. Fluorescence emission spectra were collected with integration time 5 s. The presented spectra were corrected to the reference measurement (empty metal spoon cleaned by ethanol).

### 2.7 Measurement of OJIP chlorophyll fluorescence kinetics

The chlorophyll fluorescence induction kinetics, OJIP transients (Stirbet and Govindjee, 2011; Kupper et al., 2019), of 4-day-old *Arabidopsis* seedlings (*WT Col-0*) were measured directly on the Petri plate with hand-held fluorometer AquaPen AP 110/P (Photon Systems Instruments, Czech Republic). The OJIP data were collected with the blue illumination of 470 at 1000 µmol.m^-2^s^-1^ light intensity of saturating pulse. The detection is defined by optical filters to 667 – 750 nm. The presented data were corrected to the dark offset of the instrument. Otherwise, the non-normalized data are presented to demonstrate the difference between the signal measured from the plant seedlings versus the reference measurement on pure agar-solidified MS media background.

## 3 Methods

### 3.1 iReenCAM: a novel tool for non-invasive real-time imaging of chlorophyll fluorescence *in vivo*

The iReenCAM refers to a comprehensive methodological platform comprising customized HW configuration and optimized SW packages, which allow synchronized automated measurement and analysis of the acquired fluorescence image data (**Figure 1A**). The HW design of the iReenCAM system is based on the kinetic imaging fluorometer from Photon Systems Instruments (PSI, Drasov, Czech Republic). The system is compact and allows easy dark adaptation of an investigated sample. The key component of the device is a sensitive CCD camera with high resolution (1360 x 1024 pixels) that provides sufficient spatial resolution for imaging of young *Arabidopsis* seedlings. Chlorophyll biosynthesis in seedlings was monitored by detecting the fluorescence emission signal of chlorophyll, the detection of which was optimized for spectral region 680–750 nm. The detection shifted towards longer wavelengths of chlorophyll emission should exclude possible crosstalk with shorter-wavelength emission of its precursors such as protochlorophyllide (see **Supplementary Figure 2**). The iReenCam includes a motorized, software-controlled 7-positions filter wheel with filter set designed for chlorophyll fluorescence detection in region between 680–750 nm and other 6 positions available e.g. for simultaneous detection of other fluorophores in addition to chlorophyll. Finally, the iReenCAM device comprises precisely adjustable LED panels providing uniform irradiance over samples area 90 × 90 mm. Chlorophyll biosynthesis in the etiolated seedling was induced and excited by the blue LEDs with 470nm peak wavelength. However, the iReenCAM design enables integration of up to 3 different illumination colors that opens up a wide range of possibilities for chlorophyll biosynthetic pathway regulations and mutant screening. Comprehensive control module for automatic and programmable image acquisition and data management based on PlantScreen™ phenotyping SW toolbox was used for automatic user defined measurement protocol design, data acquisition and image-based parameters extraction and analysis (**Figure 1A**).

To reliably assay the early stages of chlorophyll biosynthesis, number of measuring parameters were optimized. These include the actinic light intensity and wave length, the type of the growth media, seed sowing density, seedling growth (horizontal vs vertical plate orientation), the way of intensity mask generation (see next section) and the total length of the measurement period. The result of a laborious and time-consuming optimization is the presented protocol in which the fluorescent images of vertically cultivated four-days-old etiolated seedlings are acquired in 2-minute time intervals during a 4-hour measurement period (see Materials and Equipment for detailed description). Briefly, the measuring protocol was optimized as follows. Each measurement round (lasting 2 min in our study) started with a 50 ms long pulse of blue light (470 nm) with 240 µmol m^−2^ s^−1^ followed by a complete darkness until beginning of the next round (**Figure 1B**). In our setup, the actinic light was used for both *i)* inducing the initiation of chlorophyll biosynthesis in the etiolated seedling and *ii)* for exciting the fluorescence signal used for chlorophyll level quantification. The measuring protocol was designed to ensure that minimum required light energy was delivered to the plant samples and still enough measuring timepoints are obtained to resemble in high resolution kinetic profile of chlorophyll biosynthesis upon de-etiolation. The measurement area was optimized to ensure the maximal homogeneity of actinic light intensity for sample illumination that was kept above 70% of the maximum light intensity used (**Supplementary Figure 1**).

Along with high frequency of the measurements ensuring sufficient time resolution effective image-processing pipeline was employed. The PlantScreen™ Data Analyzer software was used to evaluate the intensity of chlorophyll fluorescence signal over the region of interest (ROI) defined by specific mask. The ROI mask corresponding to the individual seedling tissue types (cotyledons, hypocotyls, roots) was assigned and used as a mask for signal quantification, taking into account fluorescence signal intensity threshold set to subtract background noise (more details in Materials and Equipment, **Figure 1C**). The mask was defined on the image taken at the time of 3 h (showing near to maximal levels of fluorescence intensity in cotyledons, **Figure 1C**) and used to extract the signal intensity in the ROI, calculating the mean intensity of all the pixels from the ROI, over the 120 measuring rounds, 4 h period of single measuring protocol respectively. The output data comprised the fluorescent intensity values for the selected ROIs from all the seedlings imaged (approx. 30 seedlings of a given genotype in one cell of the sowing grid per experiment when using a typical setup). Thus, 90+ measurements (minimum 3 cells, i.e. 90 seedlings per genotype) are used to calculate the mean value at each time point, allowing robust statistical evaluation and detection of even small differences as statistically significant (see below and Materials and Equipment section).

## 4 Results

### 4.1 iReenCAM allows analysis of chlorophyll accumulation kinetics during *Arabidopsis* de-etiolation at high spatiotemporal resolution

Cotyledons were previously shown to accumulate chlorophyll during de-etiolation (Solymosi and Schoefs, 2010). However, the hypocotyls are also partially greening during de-etiolation. Taking advantage of the high-resolution camera used in iReenCAM, we determined the chlorophyll fluorescence separately in cotyledons, hypocotyls and roots. Using the newly established approach, we show that cotyledons reveal by far the highest dynamics in the chlorophyll accumulation when compared to hypocotyls and roots (**Figure 2**).

**Figure 3:**
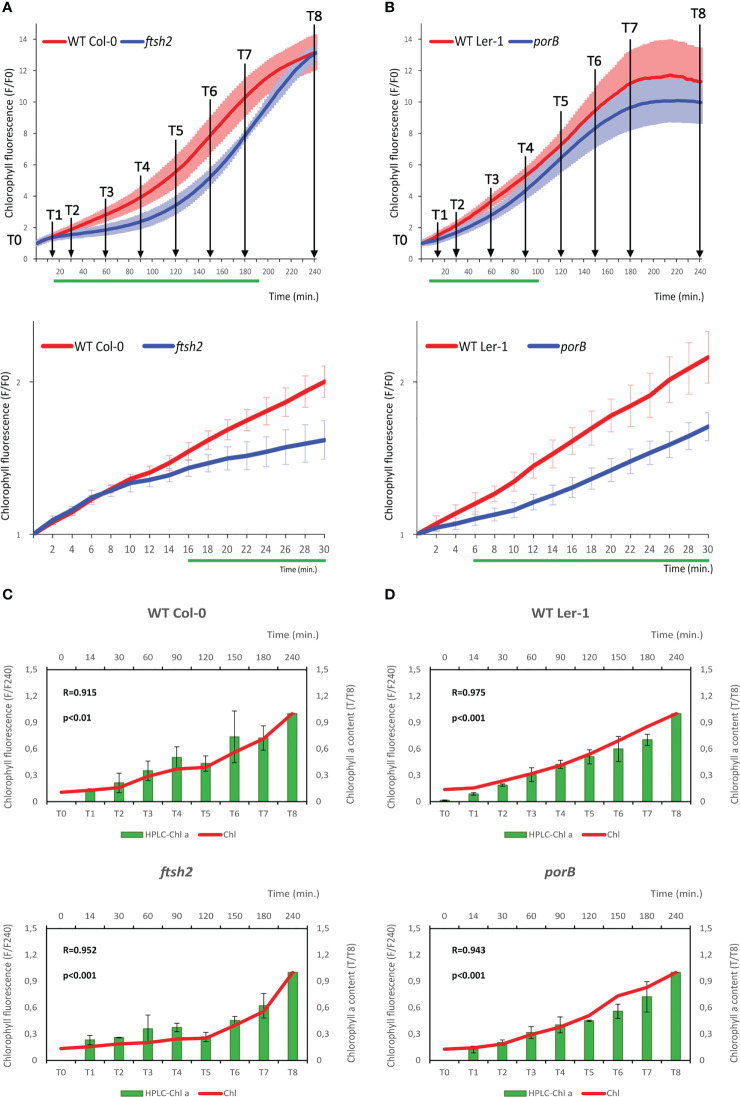
iReenCAM results tightly correlate with HPLC-measured chlorophyll *a* kinetics in both *WT* and chlorophyll biosynthesis-deficient mutants. **(A, B)** Chlorophyll accumulation in 4-days-old etiolated *WT Col-0* and *ftsh2* mutant line **(A)** and *WT Ler-1* and *porB* seedlings **(B)**. Data represent the mean values ± SD, n=9 of raw data (fluorescence F) normalized to the mean fluorescence value at T0 (F0). The green lines under the x axis depict time intervals with significantly different values between WT and the mutant (P<0.05). The time points (T0 to T8) used for seedlings collection and HPLC analysis **(C, D)** are designated in the charts. **(C, D)** Results of HPLC analysis of chlorophyll content in T0-T8 in *WT Col-0* and *ftsh2* mutant **(C)** and *WT Ler-1* and *porB* mutant **(D)** seedlings. Data are the mean ± SD, n=9 of raw data (mg/g of fresh weight normalized to T8). Chlorophyll fluorescence measured by iReenCAM (normalized to T240) positively correlates with chlorophyll *a* content measured by HPLC analysis for both *WT* and mutant backgrounds used; R-value represents Pearson’s correlation coefficient.

To address the sensitivity and specificity of the newly developed instrument and protocol, the chlorophyll content was assayed in de-etiolated seedlings of mutants previously shown to be affected in chlorophyll biosynthesis. Two groups of mutants were analyzed. The first group includes lines deficient in the chloroplast FtsH protease (Zaltsman et al., 2005). Mutation in the *FtsHs* affects chloroplast development and maintenance, leading to leaf variegation phenotype in *ftsh2* and *ftsh5* knockout lines, whereas single *ftsh1* and *ftsh8* lines reveal no obvious phenotypic alterations (Sakamoto et al., 2003). The second group comprises *Arabidopsis* mutant lines affected in the *NADPH-PROTOCHLOROPHYLLIDE OXIDOREDUCTASE* (*POR*) genes [*porB* and *porC*; (Frick et al., 2003)], coding for a key enzyme of chlorophyll biosynthetic pathway catalyzing light-dependent protochlorophyllide conversion to chlorophyll. In *ftsh2, ftsh5* and *porB* lines statistically significant reduction of chlorophyll biosynthesis was detected (**Figures 3A, B**; **Supplementary Figure 3**). As expected from the previously described expression profiles and the role of selected genes in the chlorophyll biosynthesis (see Discussion), the reduction of chlorophyll fluorescence was visible throughout the majority of the assayed time course in the *ftsh2*, while *ftsh5* and *porB* mutants were affected specifically in the early stages of de-etiolation.

To confirm that the iReenCAM-measured fluorescence corresponds to the chlorophyll content, we quantified the chlorophyll *a* and *b* accumulation in the same seedlings imaged with iReenCAM after methanol extraction by HPLC in selected time intervals (**Figure 3**). As expected, (based on the spectral properties of the filter used, see Materials and Equipment and **Supplementary Figure 2**), the results of our measurements show a highly significant correlation between the iReenCAM-measured fluorescence and HPLC-measured chlorophyll *a* content in both *ftsh2* and *porB* mutants and corresponding *WT* backgrounds (*Col-0* and *Ler-1*, respectively; **Figures 3C, D**). Slightly lower, but still highly significant correlation was seen between the fluorescence signal and HPLC-measured chlorophyll *b* content. Because of the spectral properties of the filter used (680–750 nm), the chlorophyll a to chlorophyll b ratio cannot be inferred from the fluorescence signal (**Supplementary Figure 4**).

In order to correlate the kinetics of the chlorophyll accumulation with the levels of protochlorophyllide, the POR substrate, we quantified the protochlorophyllide content using HPLC in the samples taken for the chlorophyll accumulation. As can be seen in **Supplementary Figure 5**, in the *WT Col-0* there is apparent fast decrease of the dark phase-synthesized protochlorophyllide content, reaching its minima at approx. 60 min (T3) after illumination, followed by a short lag phase. An increase due to light-induced protochlorophyllide biosynthesis is detectable at approx. 120 min (T5). Interestingly, in spite of comparable protochlorophyllide levels in *Col-0* and *ftsh2* at T0, we observed drop in protochlorophyllide and chlorophyll biosynthesis in the *ftsh2* in comparison to *Col-0* (**Supplementary Figure 5B**), suggesting possible role (direct or indirect) of FTSH2 in the light-induced protochlorophyllide production.

Finally, to characterize chlorophyll-protein complexes formation and their putative impact on variable fluorescence formation, the chlorophyll fluorescence spectra at 77 K were measured during 4 h of de-etiolation induced by our iReenCAM protocol. At early timepoints (T0, **Supplementary Figure 6**), the fluorescence spectroscopy revealed two distinct emission maxima at approx. 630 and 654 nm, corresponding to the free protochlorophyllide and photoactive protochlorophyllide (protochlorophyllide:LPOR : NADPH) complexes, respectively (Franck et al., 2000). During the first 10 minutes of illumination, the presence of a broad 675-nm peak represents chlorophyll after the Shibata shift (Sandoval-Ibanez et al., 2021). At 10 and 30 min after illumination, we observed peak at 679 nm, representing the unbound chlorophyll [i.e. the chlorophyll that has not yet been associated with photosystems complexes (Rudowska et al., 2012)], gradually growing at the expense of the photoactive protochlorophyllide (654 nm). In the further time course of de-etiolation, the maximum of unbound chlorophyll peak (679 nm) shifted closer towards 683 nm and another peak appeared as its shoulder at approximately 695 nm, being clearly recognizable at 180 min. This spectral signature indicates formation of PSII core antennae complexes including CP43 and CP47 proteins (Lamb et al., 2018). At 150 min of illumination, the 680 - 683 nm peak became more pronounced while the protochlorophyllide peak reemerged, well corresponding to our HPLC data, suggesting increase of light-induced protochlorophyllide biosynthesis (**Supplementary Figure 5A**). At 60 min after illumination, a new wide peak started arising at emission maxima between 720 and 760 nm, previously attributed to assembled PSI-LHCI peripheral antenna complexes (Andreeva et al., 2003; Rudowska et al., 2012). During the later stages between 150 and 240 min, the broad red-shifted peak between 720 and 760 nm was growing steadily suggesting an increasing proportion of assembled light harvesting complexes (both the PSI-LHCI and PSII-LHCII).

To get insight into the possible photosynthetic activity of the observed photosystem complexes within the early de-etiolation, the chlorophyll fluorescence induction kinetics, OJIP transients (Stirbet and Govindjee, 2011; Kupper et al., 2019) were measured. As can be seen in **Supplementary Figure 7**, our data show absence of detectable photosynthetic activity during the first 4 hours of de-etiolation in our experimental setup.

To sum it up, iReenCAM allows sensitive, non-destructive and specific detection of chlorophyll biosynthesis *in vivo*. While formation of the photosystem complexes was observed in our experimental setup, no photosynthetic activity was detectable during the first 4 h of de-etiolation.

### 4.2 Cytokinin and ethylene treatments affect chlorophyll dynamics during early de-etiolation

**Figure 4:**
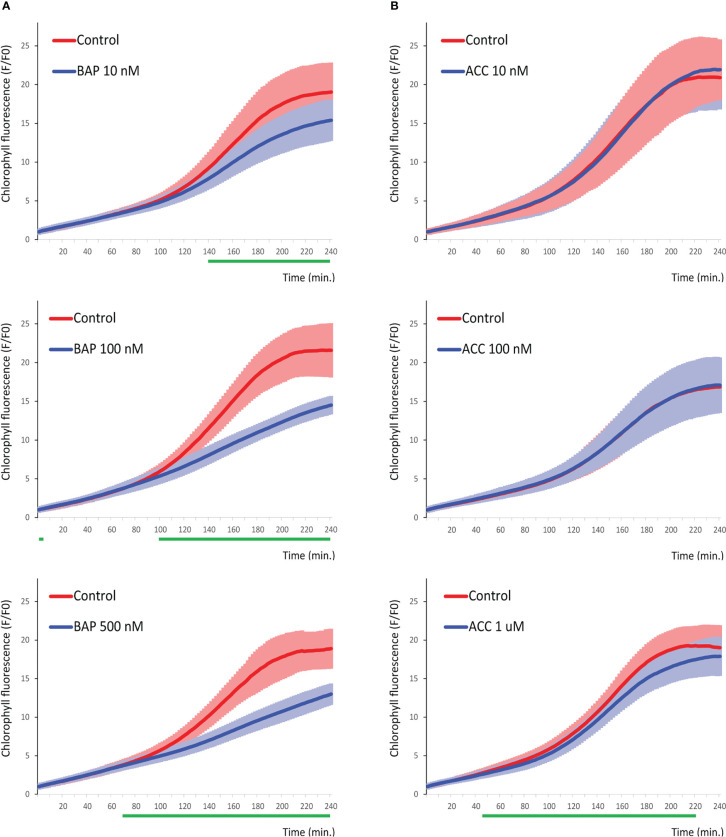
Chlorophyll dynamics is negatively regulated in a dose-dependent way by cytokinin and ethylene during the late phase of de-etiolation. Chlorophyll accumulation in 4-days-old etiolated seedlings grown on the media supplemented by **(A)** 10 nM, 100 nM, and 500 nM BAP and **(B)** 10 nM, 100 nM, and 1 uM ACC. Data represent the mean values ± SD, n=9 of raw data (fluorescence F) normalized to the mean fluorescence value at T0 (F0). The green lines under the x axis depict time intervals with significantly different values between *WT* and the mutant (P<0.05).

Cytokinin and ethylene are plant hormones that regulate essential biological processes of plant development including chlorophyll biosynthesis during seedling de-etiolation (Liu et al., 2017). To examine the effects of both regulators on the real-time measured dynamics of the chlorophyll accumulation during early stages of de-etiolation, we imaged the plants treated with exogenous cytokinin (6-benzylaminopurine, BAP) and 1-aminocyclopropane-1-carboxylate (ACC), the rate-limiting ethylene biosynthesis precursor by iReenCAM. Wild-type (*Col-0*) seedlings separately grown on mock- and hormone-supplemented plates were assayed in parallel. When compared with mock-treated controls, we observed that both growth regulators impact the dynamics of chlorophyll accumulation in a concentration-dependent manner (**Figure 4**). The cytokinins show a dose-dependent negative effect that can be observed at even very low (10 nM BAP) concentration, significantly suppressing chlorophyll biosynthesis in the later stages (after 140 min). Application of higher, 100 and 500 nM BAP cytokinin doses attenuates chlorophyll accumulation earlier, i.e. after 100 and 70 min, respectively after seedlings’ exposure to the light (**Figure 4A**). Compared to that, the response to ethylene seems to be less distinct with both 10 nM and 100 nM ACC treatments revealing no significant changes in chlorophyll kinetics compared to control (**Figure 4B**). The only significant effect was observed upon application of high (500 nM and 1 µM ACC) dose, leading to significantly decreased rate of chlorophyll biosynthesis in less than 1 h after initiation of de-etiolation (**Figures 4B**; **Supplementary Figure 8**). Based on these observations, 100 nM of BAP and 1 µM of ACC was selected for further experiments.

### 4.3 Arabidopsis type-B response regulators ARR1 and ARR12 are negative regulators of early chlorophyll biosynthesis

**Figure 5:**
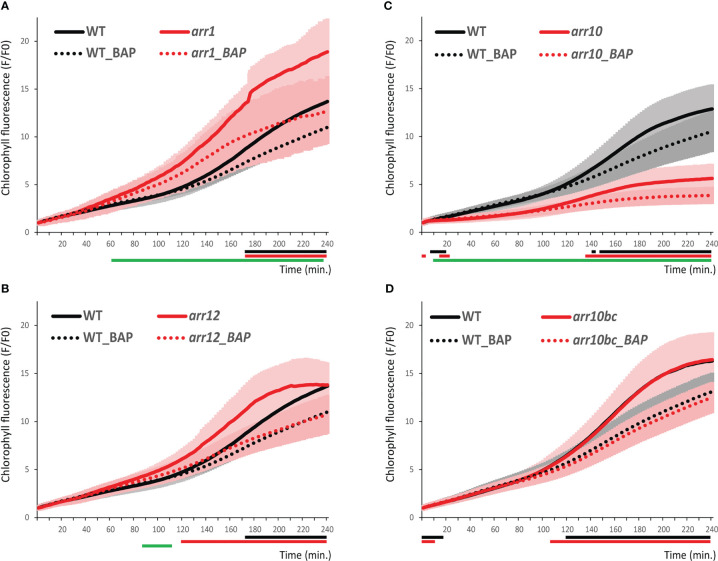
Chlorophyll kinetics in wild-type and type-B ARR mutants. Chlorophyll accumulation in *WT Col-0* and *arr1* **(A)**, *arr12* **(B)**, *arr10* **(C)**, and *arr10* backcrossed (*arr10bc*, **(D)** mutant lines. Data represent the mean values **±** SD, n=9 of raw data (fluorescence F) normalized to the mean fluorescence value at T0 (F0). Lines under the x axis (black for *WT* and red for mutant) indicate the time intervals with significantly different values in pairwise comparison between the non-treated and BAP-treated seedlings of a given genotype. The green lines under the x axis depict the significantly different values (P<0.05) the corresponding time intervals between mock-treated WT and mutant line.

ARR1, ARR10, and ARR12 were shown to be the main type-B response regulators mediating cytokinin-regulated gene expression, acting as the central hub of cytokinin signal transduction (Ishida et al., 2008). ARR1, ARR10 and ARR12 control the cytokinin-dependent gene expression including the chlorophyll biosynthetic genes (Cortleven and Schmulling, 2015; Cortleven et al., 2016). To determine the possible role of ARR1, ARR10 and ARR12 in the dynamics of chlorophyll accumulation during early stages of de-etiolation, we inspected the corresponding *arr1*, *arr10* and *arr12* mutant lines growing in the absence or presence of cytokinin by iReenCAM (**Figure 5**). Compared to WT, both *arr1* and *arr12* mutants revealed faster chlorophyll accumulation, although the difference seen in the *arr1* mutant was more distinct. In contrast to that, *arr10* mutant exhibited reduced rate of chlorophyll biosynthesis, reaching only up to 70% of WT levels during first 40 min of the measurement. This associated with pale leaf phenotype of the rosette leaves in soil-grown *arr10* line (**Supplementary Figure 9**). However, the pale-leaf phenotype disappeared in the back-crossed *arr10* line (*arr10bc*), suggesting presence of another mutation being responsible for the chlorophyll deficiency observed in the *arr10-5*. In line with that, the *arr10bc* line demonstrates normal color of the rosette leaves and no difference was detectable between the *arr10bc* and *WT* in the iReenCAM-measured chlorophyll dynamics (**Figure 5D**; **Supplementary Figure 9**).

### 4.4 The regulators of ethylene signaling have a contrasting effect on chlorophyll biosynthesis

**Figure 6:**
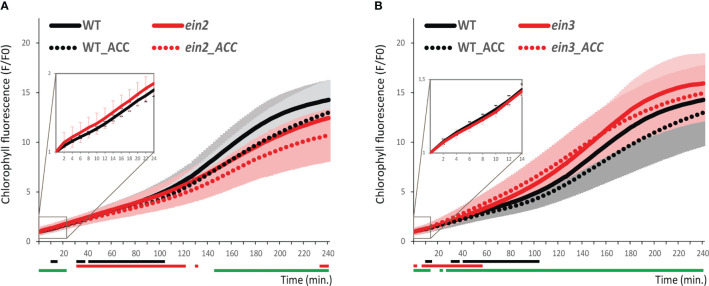
Chlorophyll kinetics in wild-type and ethylene-insensitive mutants. Chlorophyll accumulation in 4-days-old etiolated seedlings in *WT Col-0* compared to **(A)** *ein2* and **(B)** *ein3*. Data represent the mean values ± SD, n=9 of raw data (fluorescence F) normalized to the mean fluorescence value at T0 (F0). Lines under the x axis (black for wild-type and red for mutant) indicate the time intervals with significantly different values (P<0.05) comparing non-treated and ACC-treated genotypes. The green lines under the x axis depict the significantly different values (P<0.05) the corresponding time intervals between mock-treated *WT* and mutant line.

In our iReenCAM approach, the lines deficient in two key positive regulators of ethylene signaling EIN2 and EIN3 demonstrate a contrasting effect on chlorophyll biosynthesis (**Figure 6**). Compared to *WT*, the chlorophyll biosynthesis in the *ein2* mutant is attenuated in the later stages (starting at approx. 150 min after illumination) under both control and ACC-treatment conditions. Compared to that, the *ein3* line shows a slightly higher chlorophyll accumulation rate than *WT*, indicating EIN3 being a negative regulator of chlorophyll biosynthesis during de-etiolation. However, in the early stages, we have observed effects mirroring the aforementioned differences observed later after illumination in both lines, i.e. negative effect of EIN2 while positive effect in EIN3. The early stages are also revealing weak but significant sensitivity of both *ein2* and *ein3* mutants to ACC treatments that is lost in the later stages, very probably as a result of higher variability (**Figure 6**).

## 5 Discussion

### 5.1 iReenCAM allows fast and reliable chlorophyll detection with high temporal resolution

Based on the previously published data on the kinetics of the early stages of chlorophyll biosynthesis observed in the de-etiolating seedlings exposed to white light, the first burst of chlorophyll formation is followed by a lag phase due to the consumption of a dark-phase accumulated protochlorophyllide. Then, after transcriptional initiation of genes for ALA and the downstream pchlide biosynthesis, the exponential phase takes place (Virgin et al., 1963; Kobayashi and Masuda, 2016). In our setup, employing the actinic light of high intensity (470 nm, 240 µmol m^−2^ s^−1^) for both chlorophyll fluorescence excitation and de-etiolation initiation, the first fast phase is rather short (approximately 10 min), followed by slightly slower (lag) phase, associated with reaching the minima of dark-synthesized protochlorophyllide (approx. 30 min after illumination). The exponential phase associated with the initiation of the newly synthesized protochlorophyllide starts at around 90 min and 120 min in *Ler-0* and *Col-0*, respectively, followed by another lag phase at approx. 4 h after the de-etiolated seedling irradiation. That time interval corresponds to the first thylakoids formation (Heyes et al., 2021). Early formation of chlorophyll-protein complexes were observed after 2-3 hours of de-etiolation in bean and *Arabidopsis* seedlings (Kowalewska et al., 2016; Sandoval-Ibanez et al., 2021) and probably less than 8 h in cucumber (Skupien et al., 2017). Our data from the 77 K fluorescence spectroscopy confirm these findings, implying assembly of photosystem complexes as soon as 60 min after initiation of de-etiolation using iReenCAM. However, no photosynthetic activity was detectable in parallelly performed OJIP transients assay. Thus, during the first 4 h after initiation of chlorophyll biosynthesis used in our study, the emitted fluorescence corresponds to the amount of newly biosynthesized chlorophyll (as could be seen by the high correlation of the normalized fluorescence and HPLC data) and is not biased by the photosynthesis energy consumption (i.e. the variable chlorophyll florescence is not present), as it is the case later after seedling illumination (Pipitone et al., 2021). However, if the extended time period of photomorphogenesis is assayed by iReenCAM, the level of photosystems assembly and possible effect of photosynthesis on the overall fluorescence levels should be tested.

In *Arabidopsis*, there are three distinctly regulated *POR* genes: *PORA*, *PORB* and *PORC*. While being structurally related, the genes are expressed at different stages of de-etiolation. *PORA* gene is expressed in young etiolated seedlings, but it is strongly repressed by light early after initiation of de-etiolation, whereas the *PORC* transcript accumulates only upon illumination. Compared to that, the *PORB* transcript is detectable in both etiolated and light-grown seedlings (Armstrong et al., 1995; Su et al., 2001; Paddock et al., 2010). Using the iReenCAM system, we were able to detect significant chlorophyll biosynthesis defects in *porB* mutants during first 100 min. of the de-etiolation. In case of *porC* line, there is tendency for lower chlorophyll accumulation during later stages (exponential phase starting at around 60 min after illumination); nonetheless because of the higher variability, the differences are statistically insignificant.

FtsH is a membrane-bound ATP-dependent metalloprotease, mediating protein quality control by degrading the photosystem II reaction center protein D1, being damaged by the absorbed light energy. However, FtsH seems to fulfill also other roles, including ROS-mediated chloroplast to nucleus retrograde signaling, biosynthesis of the phostosystem I or thylakoid membrane biogenesis [reviewed in (Kato and Sakamoto, 2018)]. In *Arabidopsis*, FtSH forms heterohexameric complexes, consisting of type A (AtFtsH1 and VAR1/AtFtsH5) and type B (VAR2/AtFtsH2 and AtFtsH8) subunits (Sakamoto et al., 2002; Sakamoto et al., 2003; Yu et al., 2004). The loss of both A or B subunits is seedling lethal, while lines deficient in VAR1/AtFtsH5 or VAR2/AtFtsH2 demonstrate leaf variegation phenotypes (Yu et al., 2004; Zaltsman et al., 2005). Among all the tested *ftsh* mutants, the significant differences we observed in case of *ftsh2 and ftsh5*, the latter showing smaller differences in the early stages after illumination (20-80 min). That reflects well the highest abundance of FTSH2 compared to the other members of the family, which also correlates with the severity of the variegated mutant phenotypes (Adam et al., 2005). Our results are also in line with recent findings, suggesting the role of VAR2/AtFtsH2 in the accumulation of photosynthetic proteins and cotyledon greening during *Arabidopsis* de-etiolation (Wang et al., 2022). However, while Wang et al. (2022) observed defects in chlorophyll accumulation 6 h after de-etiolation initiation of *var2-5* seedlings, iReenCAM measurements allowed us to detect first changes as soon as 16 min. after illumination, leading to more pronounced first lag phase and delayed exponential phase associated with disturbed light-induced protochlorophyllide formation. This is implying the role of FTSH2 very early in the photocatalysis-induced de-etiolation but also so far uncharacterized importance of FTSH2 in the later stages of light-induced chlorophyll biosynthesis.

Altogether, our findings clearly underline the importance of sufficient temporal resolution provided by the iReenCAM analysis for the study of the highly dynamic process of plant de-etiolation.

### 5.2 Both cytokinins and ethylene control early stages of de-etiolation

Cytokinins and ethylene are important regulators of de-etiolation [for a recent review see (Liu et al., 2017). In the classical experiments, cytokinin treatment partially mimicked mutant deficient in DET1, a chromatin-associated protein and negative regulator of photomorphogenesis (Chory et al., 1991; Pepper et al., 1994). More recently, cytokinin (1 µM BAP) treatment was observed to induce acceleration of chlorophyll formation in etiolated seedlings observable after 3 hours of white light irradiation via upregulation of number of chlorophyll biosynthetic genes (Cortleven et al., 2016). Compared to that, cytokinin treatment leads to strong downregulation of chlorophyll content in the rosette leaves (To et al., 2004) and MSP signaling appeared to mediate this negative regulation (To et al., 2004; Mason et al., 2005). In line with that, cytokinins were demonstrated to maintain cell proliferation while blocking the onset of photosynthesis associated with a reduction of chlorophyll accumulation in developing leaves (Skalak et al., 2019). Similarly to cytokinins, also the effect of ethylene on chlorophyll formation during de-etiolation was found to be both positive and negative. On one hand, EIN3 upregulates the expression of *PORA* and *PORB* via direct interaction with their promoters and was demonstrated to act downstream from COP1 in the promotion of cotyledon greening during de-etiolation (Zhong et al., 2009; Zhong et al., 2010). On the other hand, ethylene strongly downregulates protochlorophyllide accumulation (Zhong et al., 2009). Furthermore, the light sensor phyB directly interacts with EIN3 in the light-dependent way, guiding thus EIN3 to proteasome-mediated degradation early during de-etiolation, suggesting negative role of EIN3 in photomorphogenesis (Shi et al., 2016). Importantly, both cytokinins and ethylene show dark- and light-specific type of response (Liu et al., 2017).

In our experimental setup, we have observed slightly negative role of ethylene in the middle and later phase during the first 4 h of the de-etiolation. Accordingly, we detected upregulation of chlorophyll fluorescence in the *ein3* mutant in the corresponding time intervals, suggesting possible negative role of EIN3 in the transcriptional regulation of genes involved in the chlorophyll biosynthesis. Interestingly, we observed the opposite type of response in case of *ein2* line, suggesting positive role of EIN2 in the chlorophyll accumulation and thus more complex role of the canonical ethylene signaling pathway in the regulation of chlorophyll biosynthesis. Compared to the C-terminal part of EIN2, acting upstream of EIN3 in the ethylene signaling, the N-terminal portion of EIN2, revealing similarity to disease-related Nramp protein transporters, was proposed to mediate the sensing of unknown signal (presumably divalent cation). The potentially ethylene-independent role of EIN2 in the early stages of de-etiolation could also be judged from our observations, suggesting retained ethylene sensitivity in *ein2*, but not in the *ein3* line. Interestingly, the N-terminal portion of EIN2 is necessary for the EIN2-mediated triple response in the etiolated *Arabidopsis* seedlings (Alonso et al., 1999). However, the mechanism underlying the contrasting responses of *ein2* and *ein3* in the chlorophyll biosynthesis remains to be identified.

Compared to ethylene, we observed much stronger negative effect of cytokinin that was apparent even at 10 nM BAP, the lowest concentration tested that usually has no or only minor effect on *WT* plants in classical bioassays including root elongation (To et al., 2004; Mason et al., 2005). Furthermore, our data imply a negative effect of *ARR1*, a considerably less pronounced impact of *ARR12*, and almost no impact of *ARR10* on chlorophyll kinetics. The negative regulation of chlorophyll formation by both *ARR1* and *ARR12* even in the absence of exogenous treatment is in a good accordance to the suppressive effects of exogenous cytokinins on chlorophyll accumulation that we see in our experimental setup, suggesting the negative role of endogenous cytokinins, too. The stimulating role of cytokinins on chlorophyll biosynthesis documented in number of other studies (Hedtke et al., 2012; Kobayashi et al., 2014; Cortleven et al., 2016) was observed upon de-etiolation induced by white light. Tight interaction between MSP and light signaling was reported [reviewed in (Skalak et al., 2021)]. Thus, the light-induced phytochrome signaling, probably only partially activated in our assay using the blue (470 nm) actinic light, might be responsible for the observed differential response. The possible role of interaction between phy-mediated light signaling and MSP is recently under intense investigation in our lab.

Worth of note, the presence of pale leaf phenotype observed in the *arr10-5* mutant, previously used in number of studies (Argyros et al., 2008; Hill et al., 2013; Cortleven et al., 2016) is apparently due to existence of another mutation being not associated with *ARR10*. That is clearly highlighting the need of careful genotyping and rigorous confirmation of the causal link between the given genotype and observed phenotypical aberrations, particularly considering the previously demonstrated functional importance of tetrapyrrole biosynthesis not only for light, but also cytokinin signaling (Dobisova et al., 2017).

### 5.3 Conclusions and future outlines

Here we introduced iReenCAM as a tool allowing detailed quantification of the chlorophyll biosynthesis with high spatial and time resolution during early stages of seedling de-etiolation. The non-invasive fluorescence-based approach has a potential to establish the light-induced de-etiolation assay as a new phenotyping approach, suitable for fast, cheap, reliable, statistically robust and well quantifiable characterization of number of mutant, transgenic lines and/or treatments. After integration into (semi)automated systems, the iReenCAM might be useful in the screening for new mutants deficient in time- and spatial-specific responses, thus providing us with novel insights into the highly dynamic response of seedling de-etiolation, one of the critical stages in the plant life cycle with high applied research potential.

## Data availability statement

The original contributions presented in the study are included in the article/**Supplementary Material**, further inquiries can be directed to the corresponding author/s.

## Author contributions

TD, ZB, KP, MT and JH conceived the research, VB, TD, ZB, KP, JS, JP, RK and DA performed the experiments, VB, TD, ZB, KP, JP, IS, IK, JS, MT and JH analyzed and interpreted the data, VB, KP, ZB, JS and JH wrote the manuscript with input from the other authors. All authors contributed to the article and approved the submitted version.
