# Supplementary material for: iReenCAM: automated imaging system for kinetic analysis of photosynthetic pigment biosynthesis at high spatiotemporal resolution during early deetiolation
Source: Front Plant Sci. 2023 Apr 21;14:1093292. doi: 10.3389/fpls.2023.1093292 (PMC10160634; doi:10.3389/fpls.2023.1093292)
Supplement: Supplementary file 1 [file DataSheet_1.pdf]

Supplementary Material

1 Supplementary Figures

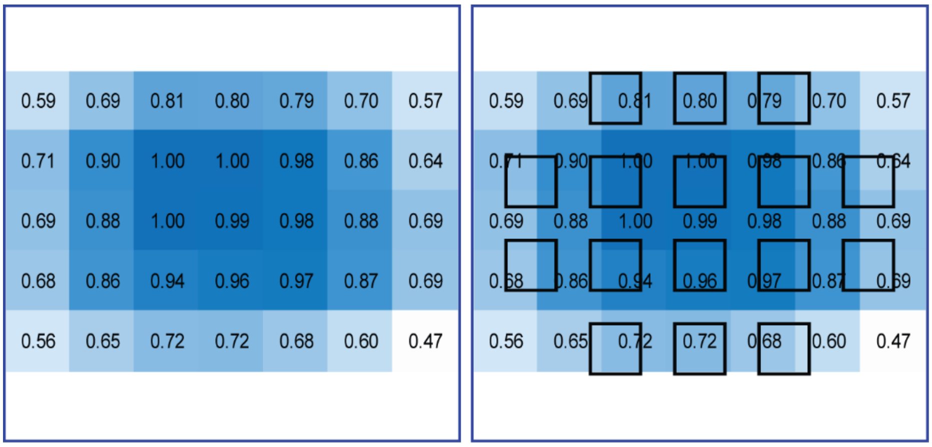

Supplementary Figure 1. Measuring area and the seed sowing grid. Left: Distribution of actinic light homogeneity (after normalization to the maximal light intensity). Right: Seed sowing grid with individual cell located in the area with ≥ 70 % (0.7) of the maximum light intensity.

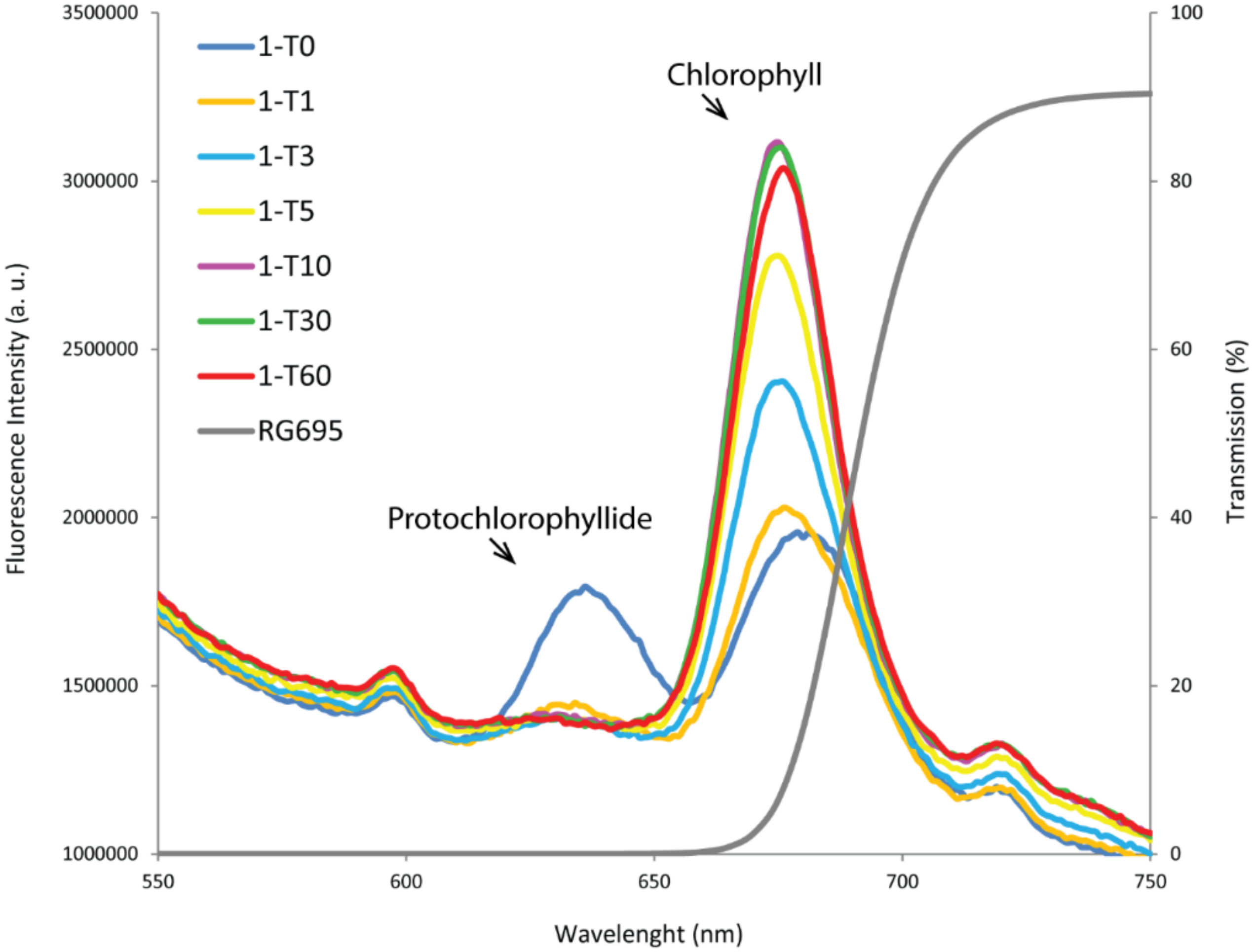

Supplementary Figure 2. Protochlorophyllide and chlorophyll emission spectra in the de-etiolating *Arabidopsis*. The spectra were obtained for intact seedlings at 0 min (T0) – 60 min (T60) after illumination. The seedlings were sown in the cuvette filled with approx. 0.5 cm thick layer of 1.5% Gelrite in buffer, wrapped in aluminum foil and placed at 4˚C in darkness for 3 days. Germination was induced under white light (150 µmol photons m^{-2} s^{-1}) for 1 h, then the seedlings were cultivated in growth chambers for 4 days in the dark at 21 ˚C. The emission spectra were captured by spectrofluorometer (Fluoromax-3, Jobin Yvon Horiba) with following settings: emission scan in the range 550-750 nm, excitation light 440nm, excitation slit = 5 nm; emission slit = 5 nm; scanning increment = 1nm, integration time = 0.1s. The whole scan lasted approximately 20s.

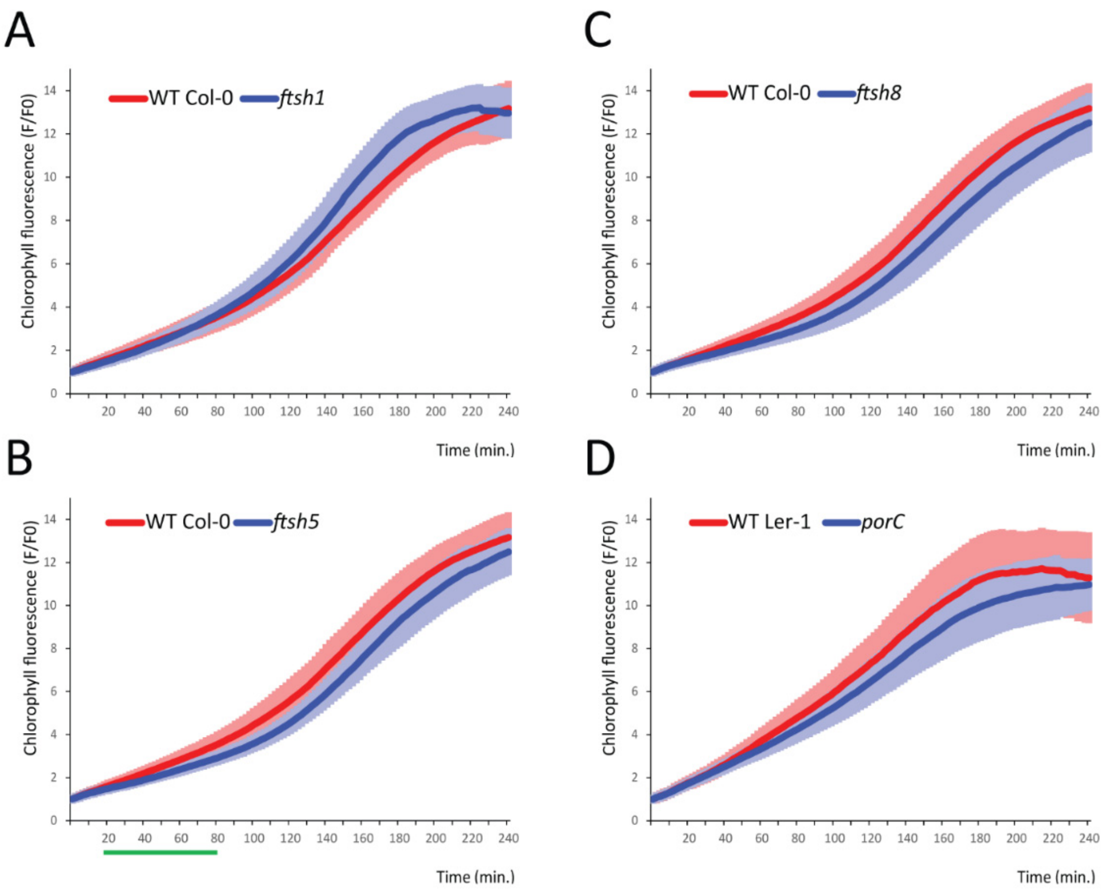

Supplementary Figure 3. Chlorophyll biosynthesis in *ftsh* mutants. Chlorophyll accumulation in 4-days-old etiolated *WT Col-0* and *ftsh1* (A), *ftsh8* (B), *ftsh5* (C) and *porC* (D) mutants. Data represent the mean values ±SD, n=9 of raw data (fluorescence F) normalized to the mean fluorescence value at T0 (F0). The green line under the x axis depict time intervals with significantly different values between *WT* and the mutant (P<0.05).

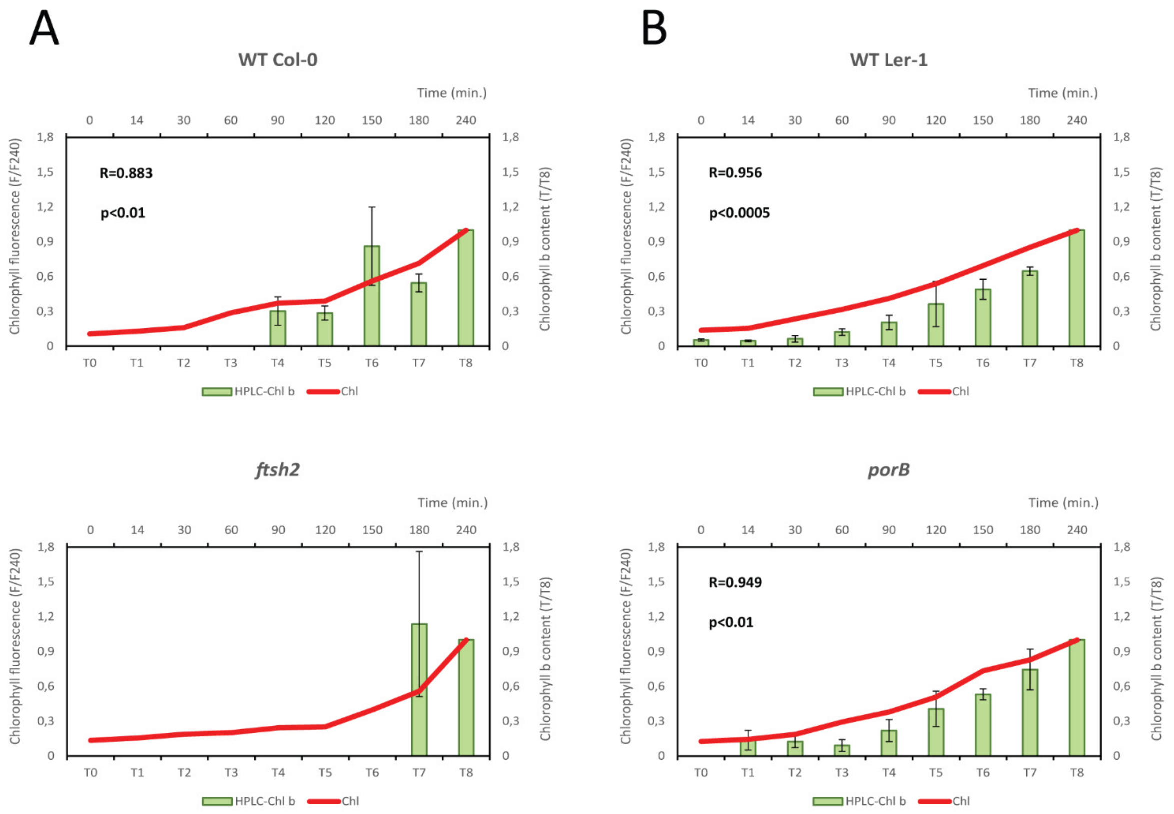

Supplementary Figure 4. iReenCAM measurements and chlorophyll *b* content determined by HPLC analysis. (A) *WT Col-0* and chloroplast biogenesis deficient *ftsh2*. (B) *WT Ler-1* and chlorophyll biosynthesis-defective *porB*. Data are the mean ±SD, n=9 of raw data (mg/g of fresh weight normalized to T8 for chlorophyll *b* measured by HPLC, chlorophyll fluorescence measured by iReenCAM normalized to T240). iReenCAM-measured fluorescence positively correlates with chlorophyll *b* content measured by HPLC analysis for both *WT* and *porB* mutant; R-value represents Pearson's correlation coefficient. The time points of seedlings collection for HPLC analysis are designated on the graphs from T0 to T8.

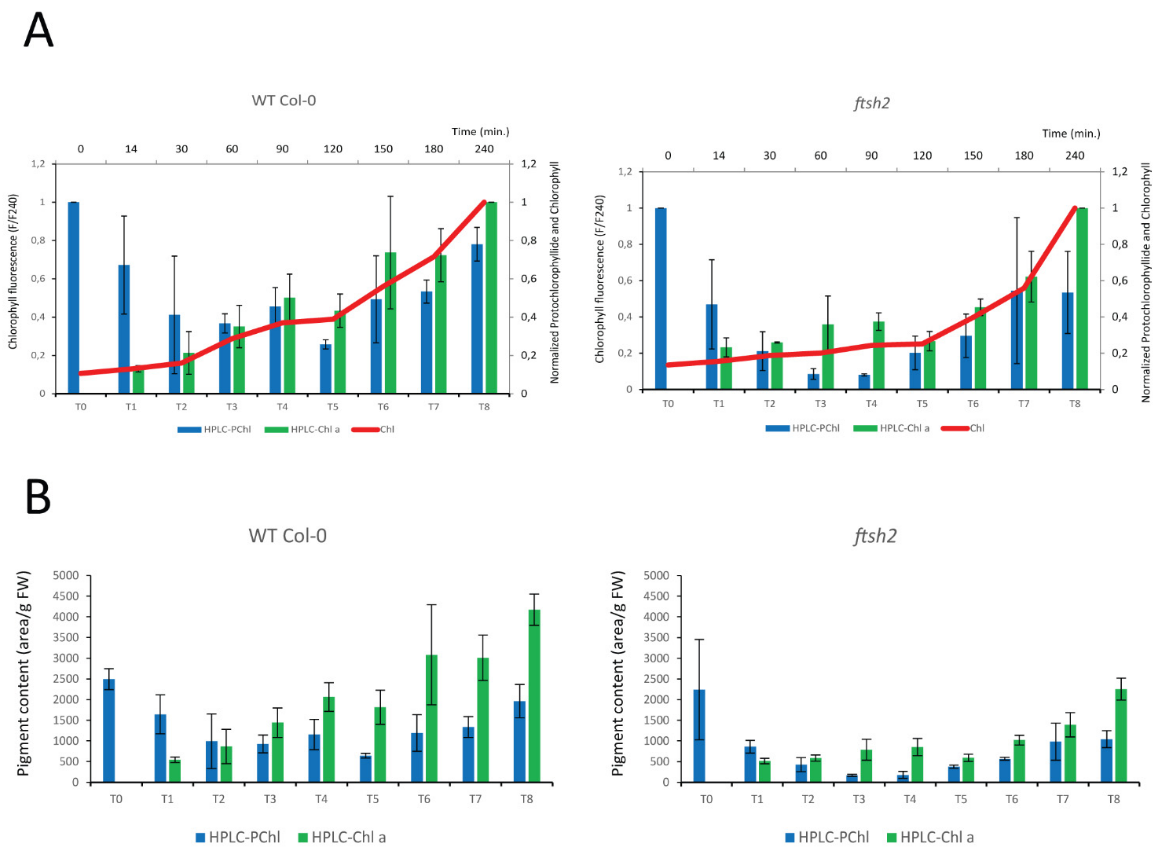

Supplementary Figure 5. Comparison of protochlorophyllide consumption and chlorophyll accumulation as measured using both HPLC and iReenCAM fluorescence. (A) HPLC-measured protochlorophyllide and chlorophyll compared to iReenCAM-measured chlorophyll fluorescence in the *WT Col-0* and *ftsh2*. Data are the mean ±SD, n=9 of raw data (mg/g of fresh weight for protochlorohyllide and chlorophyll measured by HPLC and normalized to T0 and T8, respectively; chlorophyll fluorescence measured by iReenCAM normalized to T240). (B) Non-normalized data of HPLC-measured protochlorophyllide and chlorophyll. Data are the mean ±SD, n=9.

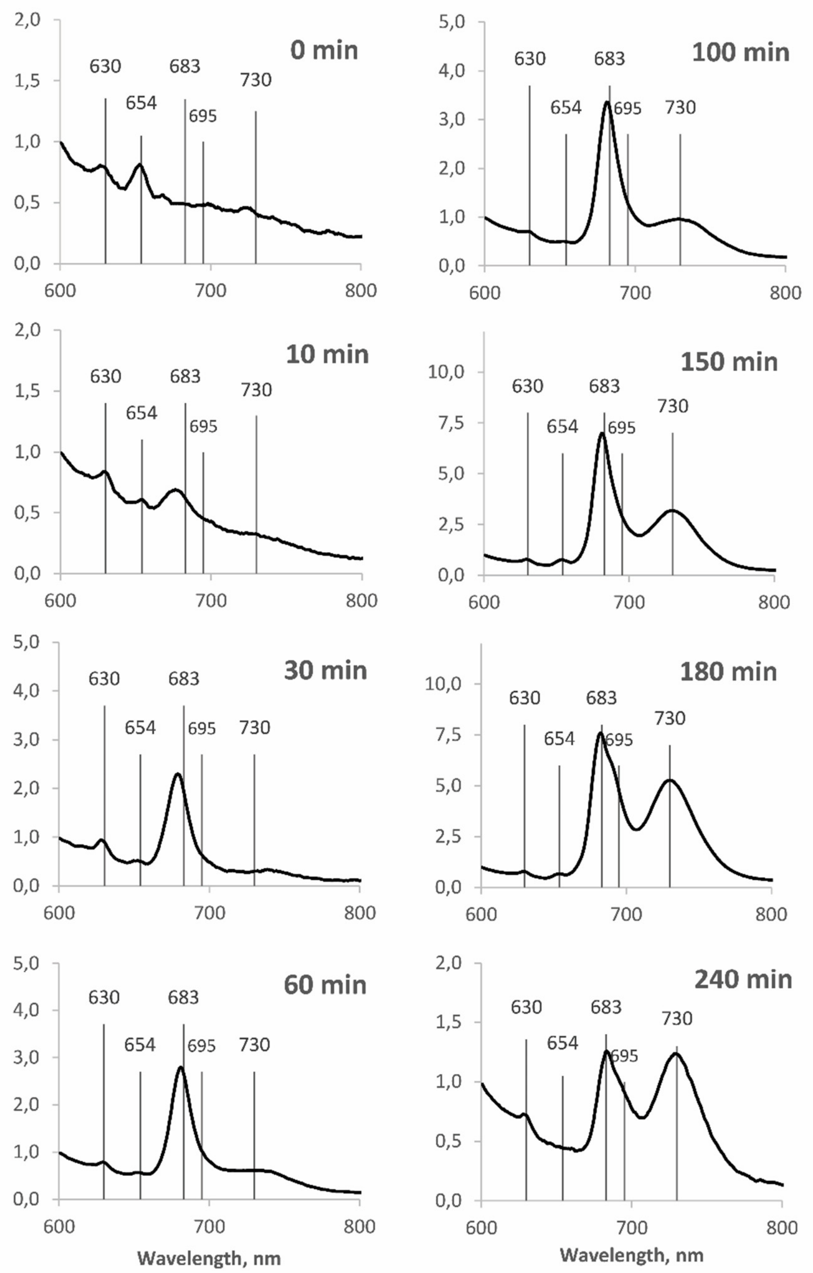

Supplementary Figure 6. Low-temperature (77K) fluorescence emission spectra (excitation at 420 nm) of 4-day-old de-etiolating *Arabidopsis WT Col-0* seedlings. The spectra were collected at given timepoints of the typical iReenCam measuring protocol as described in the Methods section. The vertical lines indicate positions of the peaks associated with free, non-associated protochlorophyllide species (630 nm), complexes containing protochlorophyllide:LPOR and protochlorophyllide:LPOR:NADPH (654 nm), LHCII complexes and CP47/CP43 PSII core antennae proteins (683 nm), CP47 (695 nm) and LHCI (730 nm) (Kowalewska et al., 2016;Lamb et al., 2018).

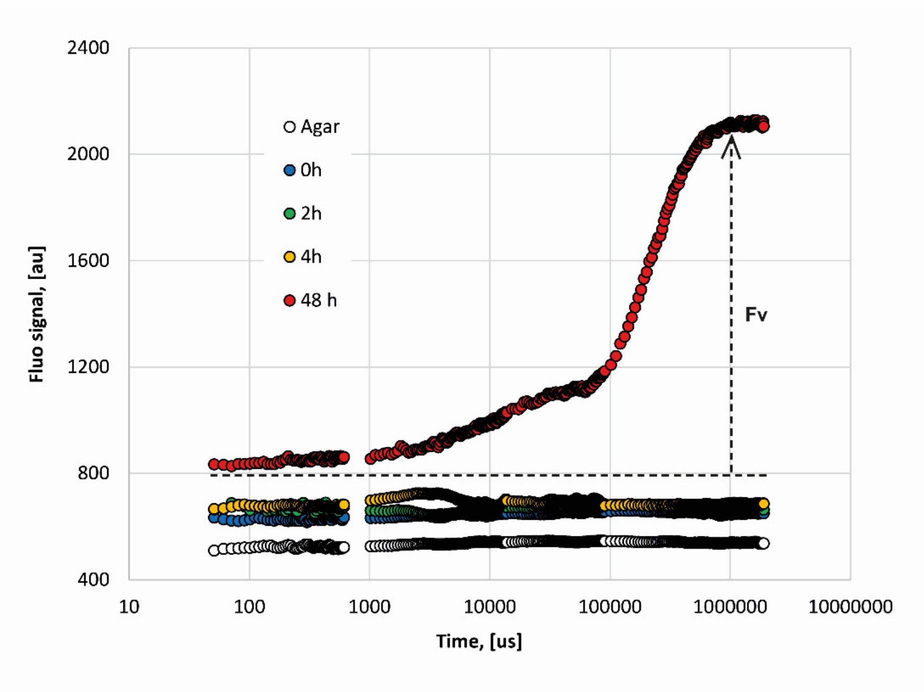

Supplementary Figure 7.

Chlorophyll fluorescence induction curves of 4-days-old etiolated *Arabidopsis WT Col-0* seedlings were determined at given time intervals after illumination using iReenCAM protocol (see Methods) and 48 hours after incubation of the same Petri plate with remaining bunch of seedlings in a growth chamber (white light, 130 umol/m2/s, 21°C/19°C at 18 h light/ 6 h dark period, respectively). Surface of agar-solidified MS media (without any seedlings) was measured as the fluorescence background. Changes in variable fluorescence (highlighted by vertical arrow) were detected only after 48 h of the incubation in growth chamber under aforementioned conditions (positive control).

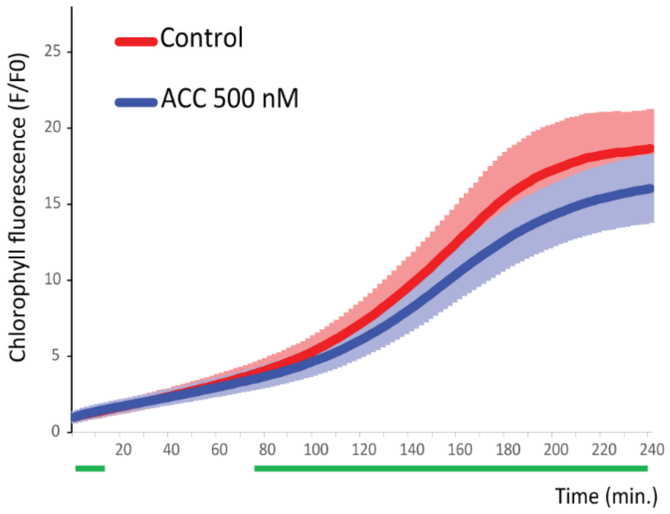

Supplementary Figure 8. Effect of ethylene (ACC) on the rate of chlorophyll biosynthesis. Chlorophyll accumulation in 4-days-old etiolated seedlings grown on the media supplemented by 500 nM ACC. Data represent the mean values ±SD, n=9 of raw data (fluorescence F) normalized to the mean fluorescence value at T0 (F0). The green lines under the x axis depict time intervals with significantly different values between *WT* and the mutant (P<0.05).

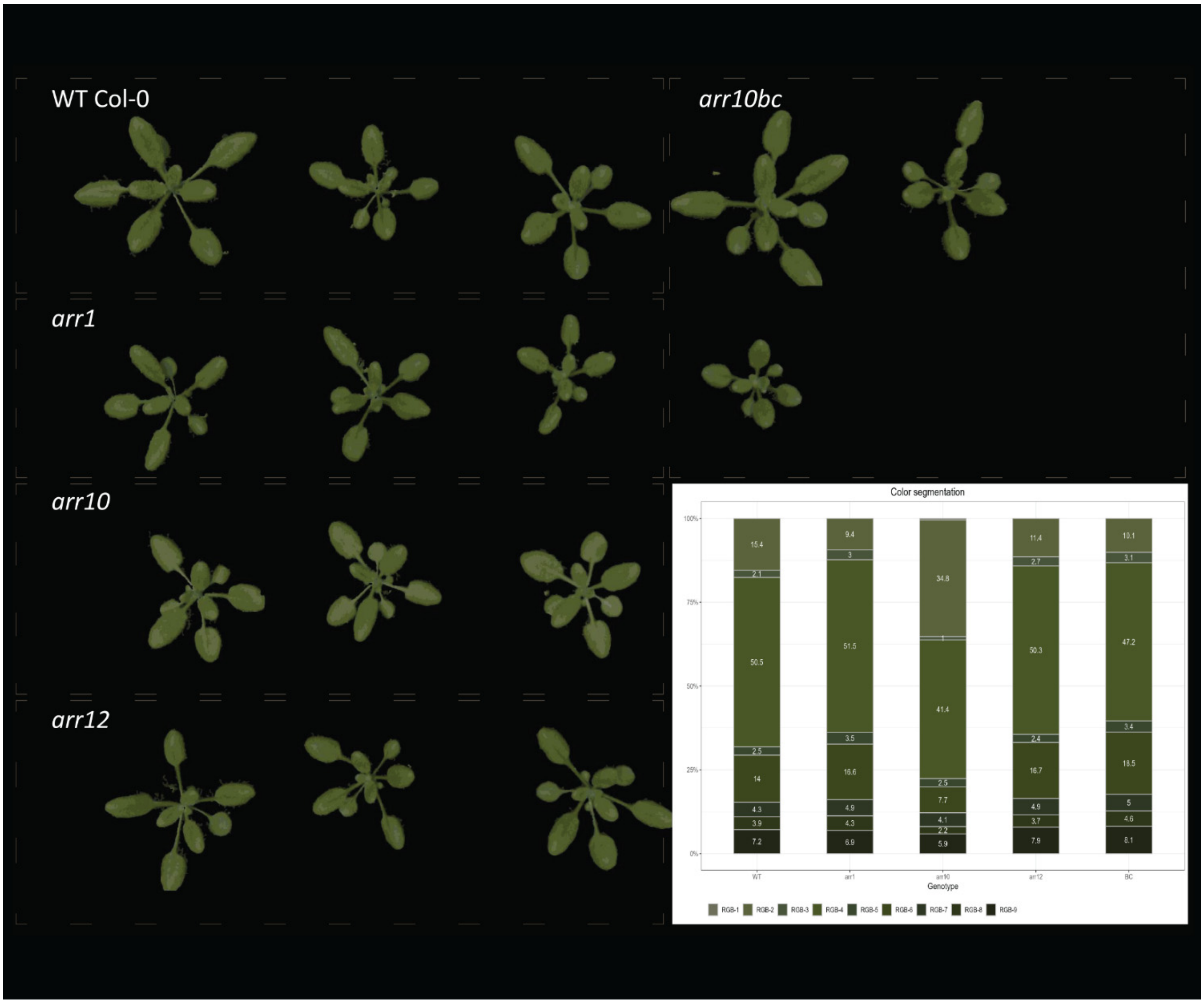

Supplementary Figure 9. Plant morphology of *Arabidopsis* response regulators mutants. Plant phenotypes of the indicated genotypes grown under long day (16-h light/8-h dark) conditions. Note the pale-leaf phenotype of the *arr10-5* line being absent in the back-crossed line (*arr10bc*).

Supplementary References

Kowalewska, L., Mazur, R., Suski, S., Garstka, M., and Mostowska, A. (2016). Three-Dimensional Visualization of the Tubular-Lamellar Transformation of the Internal Plastid Membrane Network during Runner Bean Chloroplast Biogenesis. *Plant Cell* 28**,** 875-891.

Lamb, J.J., Rokke, G., and Hohmann-Marriott, M.F. (2018). Chlorophyll fluorescence emission spectroscopy of oxygenic organisms at 77 K. *Photosynthetica* 56**,** 105-124.
